# Effects of an interactive CD-program on 6 months readmission rate in patients with heart failure – a randomised, controlled trial [NCT00311194]

**DOI:** 10.1186/1471-2261-6-30

**Published:** 2006-06-24

**Authors:** Agneta Björck Linné, Hans Liedholm

**Affiliations:** 1 Drug and Therapeutics Committee, Malmö University Hospital, MFC, Ing 59, S-205 02 Malmoe, Sweden

## Abstract

**Background:**

Disease-management programmes including patient education have promoted improvement in outcome for patients with heart failure. However, there is sparse evidence concerning which component is essential for success, and very little is known regarding the validity of methods or material used for the education.

**Methods:**

Effects of standard information to heart failure patients given prior to discharge from hospital were compared with additional education by an interactive program on all-cause readmission or death within 6 months. As a secondary endpoint, patients' general knowledge of heart failure and its treatment was tested after 2 months.

**Results:**

Two hundred and thirty patients were randomised to standard information (S) or additional CD-ROM education (E). In (S) 52 % reached the endpoint vs. 49 % in (E). This difference was not significant. Of those who completed the questionnaire (37 %), patients in (E) achieved better knowledge and a marginally better outcome.

**Conclusion:**

The lack of effect on the readmission rate could be due to an insufficient sample size but might also indicate that in pharmacologically well-treated patients there is little room for altering the course of the condition. As there was some indication that patients who knew more about their condition might fare better, the place for intensive education and support of heart failure patients has yet to be determined.

## Background

The prevalence of heart failure has increased in the western world due to ageing of the general population and improved survival of patients with acute coronary artery disease [1]. Heart failure (HF) is a major concern for health care providers due to increasing prevalence and rising health care costs [2]. The prognosis remains poor despite improvement in survival due to treatment with ACE-inhibitors and beta-receptor antagonists [3,4]. Heart failure has a high rate of readmission and hospitalisation [5]. Some readmissions have been ascribed to patients' lack of compliance, insufficient knowledge about diet, medication and symptoms of heart failure [6-8]. Education of patients has become an important component in order to increase the patients' self-care and compliance, which might improve quality of life and reduce health care costs [9-13].

In the European (ESC) guidelines for HF intense education and counselling of patients (and other persons) is recommended [14]. However, even if the recommendations are classified as Class I, the highest of three main classes, the level of evidence for this action is grade C, the lowest of three levels.

The same pattern appears in the American guidelines [15]. In that document, it is stated that observational studies and randomized controlled trials have shown that disease-management programmes can reduce the frequency of hospitalization and can improve quality of life and functional status. However, it remains unclear which elements of disease-management programmes are crucial for success.

When these programmes are viewed in detail, their components and outcome differ. As it was commented in an editorial article, "nothing of a class effect seem to exist" [16]. However, among other things, patient education (and self-management) is emphasised in the recommendations set up by the American Heart Association [15].

Even if several systematic reviews of studies on strategies for improvement of outcomes of HF patients after discharge have been published, very little emphasis has been put on methodological issues, e.g. methods of education [17-20]. Many studies have shown that structured discharge support, especially with multidisciplinary strategies, can reduce readmission rates and in some cases even mortality. Conflicting data have also been published, e.g. in a recent nurse-directed telephonic HF management programme, no measurable benefit of disease management was found [21].

The information material in the majority of disease management programmes, as reviewed in the literature, only exist as verbal and written material. Computer-based tools seem to be relatively new [22]. In a recent review on the crucial role of patient education in HF, it is stated that education can still be improved by combining clinical experience with new technologies, such as computer-based education [23].

We have previously used an interactive CD-ROM program for heart failure patients and have shown that it is possible to increase patients' knowledge about heart failure and its treatment [24,25]. Other investigators have evaluated the user-friendliness of a similar CD-ROM based program [26]. In our previous study a significant increase of knowledge in the intervention group was shown [25]. However, none of these studies examined the clinical outcome, e.g. rate of readmission. We therefore performed a randomised multi-centre study, in order to evaluate the impact of added CD-ROM education on readmission rate or death during 6 months.

## Methods

This study aimed at education of HF patients concerning a meaningful end-point. Patients were intended to be representative of ordinary heart failure patients in mid- and small-size general hospitals in Sweden. All hospitals were community hospitals, and were supposed to follow the same clinical (national) guidelines of care of heart failure conditions. Efforts to include patients in the study remained at the discretion of participating doctors and nurses by screening consecutive in-patients treated for heart failure. To decrease other bias in selection of patients we included only clinics where no other trial in cardiology was carried out at the same time. Locally approved and standardised verbal information for heart failure patients and the use of educational material, including printed and audiovisual aids, were to be held constant during the study. The management of the patients was also restricted to a small number nurses in each hospital, giving them control of the patient flow during the study.

### Randomisation

Patients were randomised to receive conventional information/education or to additional interactive education via CD-ROM at the time of hospital discharge. Patients were randomised by telephone; hence, the sites had no access to the randomisation list. Randomisation was made by computer, and patients were stratified by clinic and in three age groups < 65, 65–75, and > 75 years.

### Inclusion and exclusion criteria

Criteria for heart failure was left ventricular ejection fraction (LVEF) < 40 % at echocardiography or at least two of the criteria pulmonary rates, peripheral oedema, a third heart sound and signs of heart failure at chest X-ray.

Patients with somatic disease or physical handicap with difficulties to communicate or handle the technical equipment and patients with only little knowledge in Swedish and patients with expected problems with compliance due to alcohol/drug abuse or major psychiatric illness, and participation in a trial, were excluded.

### Procedures

During hospital stay, the patients who fulfilled the inclusion criteria were invited to participate in the study. Patients who accepted were randomised. Informed consent was obtained in all cases.

Prior to discharge patients received the standard information, which was given at the ward. Patients randomised to the control group were then discharged to their usual care. Patients randomised to the intervention group received additional education by an interactive program on heart failure, its symptoms and treatment. This program took about 20–30 minutes. After discharge to their usual care, the patients returned to the hospital after two weeks and the CD program was repeated. The CD-program, which was displayed on a TV set from a photo CD player, has been described in detail before [24]. For the actual study the program had been changed in one respect, i.e. more explicit information on beta-blockers was given. The outline of the CD-program is shown in Table 1.

**Table 1:** Outline of content of the CD. In short – after choosing either of the main issues, as shown in the second row of the table, the patient follows the items presented in the column below. If picking a wrong alternative in response to a question, the next frame tells that it was wrong but does also mention the right answer. Then the question automatically reappears until the right answer is chosen; otherwise the program will not proceed beyond that part. After having finished one of the two main issues, the program automatically turns to the remaining issue. Figures in brackets are number of frames of the program.

Heart failure The purpose of the CD (1)	
1. The disease	2. The treatment

General (2)	Diuretics (4 special + 1 general)
Tiredness (1)	Fluid intake (1 special + 4 general)
Dyspnoea (1)	- Question (diuretics): right (1), wrong (2)
Fluid accumulation (3)	- Question (fluid intake): right (1), wrong (2)
End of first part (1)	Use of diuretics – conclusion (1)
Treatment of symptoms (1)	ACE-inhibitors (4)
Aetiology (1)	- Question: right (1), wrong (2)
Deterioration (1)	Digitalis (3)
Symptoms and signs of deterioration (2)	- Question: right (1), wrong (2)
Reasons for deterioration	Beta blockers (4)
- Pharmacological reasons (1)	- Question: right (1), wrong (2)
- Other conditions (1)	Reminder on use of diuretics (1)
Course of deterioration	End of this session (1)
End of this session (1)	
Ending message (after watching the whole CD)	

### Primary endpoint

The primary, combined endpoint of the study was difference in rate of all cause readmission and death within 6 months after discharge.

### Questionnaire

The patients' general knowledge of heart failure and its treatment were tested with a questionnaire. The questionnaire was a slight modification of a version used in an earlier study [25]. That version was developed from scratch as there were no preceding studies in Sweden. It was based on information given at the teaching sessions and the content of the CD-ROM used in that study.

The 16 questions in the questionnaire were as follows (modified after translation).

1. Mention a drug used for treatment of heart failure that can reduce swelling of the legs

2. What is the primary action of an ACE-inhibitor? Tick one alternative

Contract vessels

Dilate vessels

Improve contraction of the heart

Decrease salt and water in the body

3. Mention a drug used for treatment of heart failure that may improve your breathing

4. If your body weight goes up fast, what should you do with the dose of your diuretic drug?

5. Which drug makes you pass urine more frequently?

6. Give a reason for taking an extra dose of your diuretic drug

7. Do you measure how much you drink per day?

8. If you get sick and temporarily suffer from vomiting, what should you do with the dose of your diuretic drug?

9. Which of the following drugs is important to take exactly as prescribed? Tick one alternative

Nitroglycerin (e.g. Nitromex)

Digitalis (e.g. Lanacrist/Digoxin)

Diuretic (e.g. Impugan/Furix/Furosemid)

10. Which side-effect is likely to get from an ACE-inhibitor? Tick one alternative

Dizziness

Loss of potassium and magnesium

Upset stomach

11. At most, how much are you advised to drink daily?

12. If you get sick and temporarily suffer from diarrhoea, what should you do with the dose of your diuretic drug?

13. If your legs get swollen or the swelling increases, what should you do with the dose of your diuretic drug?

14. If you get a fever, what should you do with the dose of your diuretic drug?

15. Which of the following drugs may cause a patient to cough? Tick one alternative

Diuretic (e.g. Impugan/Furix/Furosemid)

Digitalis (e.g. Lanacrist/Digoxin)

ACE-inhibitor (e.g. Renitec/Triatec/Zestril/Capoten)

16. Which of the following drugs may cause an upset stomach and increase the risk of bleeding, e.g. if you have to have a surgical operation. Tick one alternative

Digitalis (e.g. Lanacrist/Digoxin)

Diuretic (e.g. Impugan/Furix/Furosemid)

Aspirin (e.g. Trombyl)

The questionnaire was sent by regular mail to the patients 8 weeks after randomisation. Returned questionnaires were kept sealed until the whole study was closed. A secretary, unrelated to study participants and the people responsible for the study, made a formal classification of the answers. Allocation of patients and the outcome of the study were masked.

A computerised patient administrative system, PAS, was used to verify all-cause readmissions and deaths within 6 months from discharge. Reasons for readmission or subsequent readmissions were not accounted for.

### Ethics

The investigation conforms with the principles outlined in the Declaration of Helsinki (Br Med J 1964, ii:177). The study was approved by the Committee on Research Ethics of the Lund University, Sweden.

### Statistical analyses

In an ecological study the all-cause readmission rate and deaths were shown to be 53 % of heart failure patients within 6 months [5]. At the planning of our study, there was no evidence from randomised studies using educational methods to base a reliable calculation of study size on. To increase the knowledge of a reasonable sample size for our study, we searched for useful data from published studies showing significant results of the intervention.

In a randomised study of intervention by a multidisciplinary team to prevent readmission in heart failure patients, a reduction from 67 % readmission rate in the control group to 37 % in the intervention group was shown after 90 days [12]. A post hoc calculation of power and sample size gave a power of 99 % from the actual sample size of 282 patients (nQuery Advisor^® ^5.0). A study with 80 % power would only need 100 patients.

In another study, including a clinical pharmacist in the team, the sample size of the study was 181 patients in total [27]. There were 4 cases of all-cause mortality and nonfatal heart failure in the intervention group (n = 90), and 16 cases in the control group (n = 91) during 6 months follow-up. A post hoc calculation of power gave a power of 81 % from the actual sample size. A study with 80 % power would marginally decrease the size of the sample to 175 patients. In neither of these studies an à priori calculation of power was presented.

To detect a 20 % difference in outcome between two groups with 80 % power and a significance level of 5 % (two-sided), a total sample size of 206 patients would be required (nQuery Advisor^® ^5.0) in our study.

For conventional parametric and non-parametric statistical calculations we used the statistical software StatsDirect Version 2,3,8. Tests for normality were included. For calculation of survival the Peto & Peto Wilcoxon test in the same software was used.

## Results

Much effort was spent to include clinics in this study, which was funded by non-commercial funds. Nineteen clinics were approached, but four major clinics were preoccupied with other clinical studies. The final study included clinics at the following hospitals (in alphabetical order) in Sweden: Eskilstuna, Helsingborg, Kalmar, Karlskoga, Karlskrona, Karlstad, Kristianstad, Ljungby, Nyköping, Trelleborg, Visby, Växjö, and Ystad. Two other clinics started the study but due to reorganization and introduction of new patient caring routines, they dropped out of the study. No patients from these clinics were included in the analysis. The number of screened patients per clinic ranged from 10 to 96. The first patient was randomised in February 1998 and the last patient in July 2002.

### Patient flow

**Figure 1:**
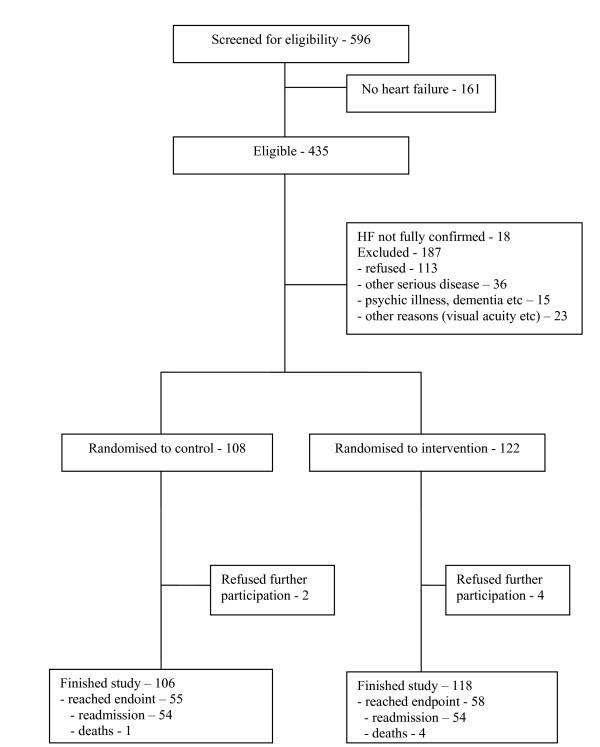
Patient distribution in the trial.

The patient flow in the study is shown in figure 1.

### Patients' characteristics

Two hundred and thirty patients were randomised (108 controls and 122 interventions).

Patient characteristics at randomisation are presented in Table 2. All patient characteristics were similar except from the use of digitalis and aspirin at discharge from hospital.

**Table 2:** Patients' characteristics at randomisation. If not otherwise stated, data is arithmetic mean and standard deviation.

**Patient characteristics**	**Controls**	**Intervention**
Number of patients	108	122
Sex (F/M)	26/82	42/80
Age, years (range)	70.8 (41–88)	70.3 (34–89)
Weight, kg	81.8 (20.2)	77.6 (17.3)
Height, cm	173.6 (8.2)	172.0 (9.6)
**Treatment at randomisation**		
Diuretics, n (%)	95 (90)	110 (90)
ACE-inhibitors, n (%)	85 (79)	99 (81)
Beta-blockers, n (%)	46 (43)	67 (55)
Spironolactone, n (%)	24 (22)	22 (18)
Digitalis, n (%)	28 (26)	54 (44)*
Aspirin, n (%)	36 (33)	60 (49)*
**Blood chemistry**		
Creatinine, μmol/L	120.1 (43.0)	111.4 (42.4)
Glucose, mmol/L (median, inter-quartile range)	5.5 (4.8–7.2)	5.65 (4.7–6.7)
Potassium, mmol/L	4.0 (0.4)	4.0 (0.4)

* Significant differences between treatment groups (Fisher's exact test), p = 0.004 and 0.016 respectively.

### Outcome measures

#### Primary endpoint

**Figure 2:**
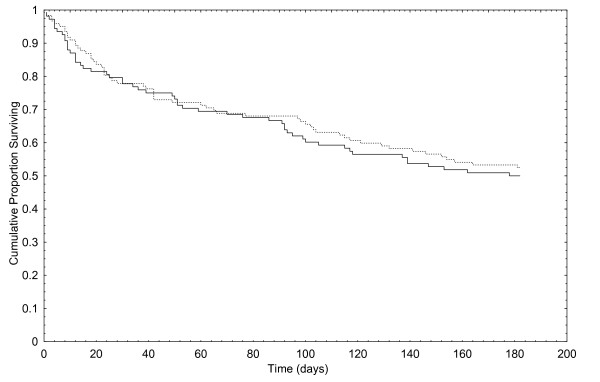
Time to first event in the intervention group (dotted line) and the control group (straight line).

The difference between the two groups was not significant. Mortality rate was low – only one patient in the control group and 4 patients in the intervention group died during the study. In the control group 55/106 (52 %) patients reached the combined end-point, in the intervention group 58/118 (49 %) patients. The Peto & Peto Wilcoxon test for survival was not significant, p = 0.592. Figure 2 shows the Kaplan-Meier curves.

#### Questionnaire

Only 82 of 224 patients, who finished the study (37 %), completed the questionnaire. Patients in the intervention group (n = 43) reached a significantly higher score (mean score 10.1 points range 4–15) than the control group (n = 39), (mean score 7.7 points, range 2–14), p = 0.004. No significant differences were seen between the groups regarding sex, age or use of drugs documented for survival in heart failure (ACE-inhibitors, beta blockers and spironolactone).

**Figure 3:**
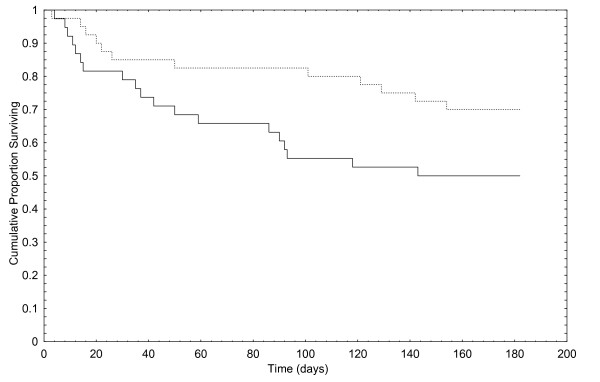
Time to first event of those in the intervention group (dotted line) and the control group (straight line) who answered the questionnaire.

A comparison of data between those who completed or did not complete the questionnaire showed no difference regarding sex, age or use of documented drugs. Analysis of primary endpoint in the sub-group who completed the questionnaire showed a borderline significant difference between the intervention group and the control group, Peto & Peto Wilcoxon test p = 0.05039. Figure 3 shows the Kaplan-Meier curves.

## Discussion

This study did not show any effect on the primary end-point of adding an interactive, CD-based education to the regular education of and information to previously hospitalised patients with heart failure. In a study with a "negative" result, one can always discuss if this is due to a type II error, i.e. the study had been underpowered. In an effort to shed light on this item, we simulated a bigger study by adding results from our previous RCT of similar design and same duration [25]. That study was only designed to test the impact of the CD program education on knowledge. Data about readmission and death from all participants (n = 108) were extracted from the files and added to the actual study. However, even after this addition, no effect on the primary end-point was detected.

Could it then be that all necessary knowledge for understanding and self-management of the condition already had been transferred to the patients and that additional education by the CD-program, even if it was repeated, was redundant? This view is not supported by the data from completed questionnaires in the actual study, where a significantly higher score was attained in the CD-educated group. However, the number of completed questionnaires was low, in spite of our ambition to follow the same management as was expressed in a recent Cochrane review [28].

The target population was representative of ordinary HF patients treated in rural and urban hospitals. The quality of the baseline care as expressed by the use of ACE-inhibitors (80 %) was high, probably a result of the impact of National Programmes in cardiology in Sweden (The Swedish National Board of Health and Welfare's Guidelines for Cardiac Care, latest version published in 2004). The management of patients in the study consisted of general nurse-based methods of care for HF patients, e.g. education, enhancing self-care, and monitoring of drug treatment.

One hundred and thirteen eligible patients refused to participate. In this trial, patients had to come twice to the CD-based education, first as in-patients, the second time at approximately 2 weeks after discharge. Returning to the hospital may have discouraged some patients to participate, in this manner recruiting less sick patients.

With regard to study planning and power for sample size calculation, two studies of interest have been published after the start of our own [29,30]. In the first study it was stated that with 80 % power the sample size should include 164 patients to detect a 23 % difference in mortality and hospital readmission due to heart failure after one year. A 26 % difference was detected after one year. In the second study (n = 280) no power calculation was given, but this study has some similarities with our own. Both included in-patients, were of the same size, drug treatment was in many aspects similar, and patients were educated before discharge from the hospital. The studies were different in other aspects. Their patients were younger (age difference 10 years) and seem to have had a more serious disease. However, readmission rate at 6 months was of the same magnitude and no statistical difference was detected.

In a recent (n = 462) study lasting one year, there was no difference regarding the primary combined endpoint (death, rehospitalisation or emergency department visit for cardiac causes or any cause) between the intervention and the control group [21]. At 6 months, the proportion of patients reaching the end-point was estimated to about 42 % in the intervention and 50 % in the control group, as judged from the survival curves published in the report. We obtained quite similar figures. In the comments to this study, the accompanying Editorial discussed the (negative) outcome of this and three earlier trials [31]. Three items were recognised as factors that could account for the divergent outcomes of similar studies: a) the target population, b) the quality of usual care, and c) the design of the management programme. Difficulties with self-management, low self-efficacy, social deprivation, and chaotic use of health care system were appointed to be more useful characteristics than traditional disease characteristics for identification of patients responsible to the management programmes.

Even if it was a post-hoc subgroup analysis based on small numbers, the better result regarding readmissions in those who completed questionnaires, gives hope that better knowledge by education and a subsequently better self-management may still work at a larger scale. This has to be tested in a formal trial where both variables are tested at the same time, which was not the case in our study. To prove that this method also may have an impact on the death rate remains, in our opinion, a much more difficult task.

Different studies continue to give conflicting results, and a full identification of prognostic factors still has to be determined. At this moment, the optimal structure and role of education and self-management programs of HF patients cannot be fully settled.

## Conclusion

Additional education of HF patients with an interactive program had no effect on readmission rate or death within 6 months after discharge. The lack of effect may be due to an insufficient sample size of the study. It might also indicate that in pharmacologically well treated patients there is relatively little room left for altering the natural course of the condition. However, as there was some indication that patients who knew more about their condition might fare better, the place for intensive education and support of HF patients has yet to be determined. The formal, controlled testing of validated methods for patient education should be encouraged. The variables should not only be knowledge but should also include patient behaviour (e.g. self-management) and a clinical outcome, such as tested in our study. Our study has made an input to the discussion of calculation of statistical power for similar studies, as was lacking at the onset.

## Competing interests

The author(s) declare that they have no competing interests.

## Authors' contributions

Both authors planned, recruited participating clinics, supervised, randomised patients, and prepared the manuscript. Both authors read and approved the final version of the manuscript.

## Pre-publication history

The pre-publication history for this paper can be accessed here:


